# Application of a Pillared-Layer Zn-Triazolate Metal-Organic Framework in the Dispersive Miniaturized Solid-Phase Extraction of Personal Care Products from Wastewater Samples

**DOI:** 10.3390/molecules24040690

**Published:** 2019-02-15

**Authors:** Providencia González-Hernández, Ana B. Lago, Jorge Pasán, Catalina Ruiz-Pérez, Juan H. Ayala, Ana M. Afonso, Verónica Pino

**Affiliations:** 1 Departamento de Química, Unidad Departamental de Química Analítica, Universidad de La Laguna (ULL), La Laguna, Tenerife 38206, Spain; mgonzalh@ull.edu.es (P.G.-H.); jayala@ull.edu.es (J.H.A.); aafonso@ull.edu.es (A.M.A.); 2 Laboratorio de Rayos X y Materiales Moleculares (MATMOL), Departamento de Física, Universidad de La Laguna (ULL), La Laguna, Tenerife 38206, Spain; alagobla@ull.edu.es (A.B.L.); jpasang@ull.edu.es (J.P.); caruiz@ull.edu.es (C.R.-P.); 3 University Institute of Tropical Diseases and Public Health, Universidad de La Laguna (ULL), La Laguna, Tenerife 38206, Spain

**Keywords:** metal-organic frameworks, pillared-layer frameworks, sample preparation, microextraction, miniaturized dispersive solid-phase extraction, personal care products

## Abstract

The pillared-layer Zn-triazolate metal-organic framework (CIM-81) was synthesized, characterized, and used for the first time as a sorbent in a dispersive micro-solid phase extraction method. The method involves the determination of a variety of personal care products in wastewaters, including four preservatives, four UV-filters, and one disinfectant, in combination with ultra-high performance liquid chromatography and UV detection. The CIM-81 MOF, constructed with an interesting mixed-ligand synthetic strategy, demonstrated a better extraction performance than other widely used MOFs in D-µSPE such as UiO-66, HKUST-1, and MIL-53(Al). The optimization of the method included a screening design followed by a Doehlert design. Optimum conditions required 10 mg of CIM-81 MOF in 10 mL of the aqueous sample at a pH of 5, 1 min of agitation by vortex and 3 min of centrifugation in the extraction step; and 1.2 mL of methanol and 4 min of vortex in the desorption step, followed by filtration, evaporation and reconstitution with 100 µL of the initial chromatographic mobile phase. The entire D-µSPE-UHPLC-UV method presented limits of detection down to 0.5 ng·mL^−1^; intra-day and inter-day precision values for the lowest concentration level (15 ng·mL^−1^)-as a relative standard deviation (in %)-lower than 8.7 and 13%, respectively; average relative recovery values of 115%; and enrichment factors ranging from ~3.6 to ~34. The reuse of the CIM-81 material was assessed not only in terms of maintaining the analytical performance but also in terms of its crystalline stability.

## 1. Introduction

Humans and living organisms are exposed to an increasing amount of emerging pollutants, with clear health issues associated [1,2]. Their low levels in environmental samples (still risky even at those levels) [1] undoubtedly require the utilization of proper analytical sample preparation techniques together with an adequate chromatographic analysis. The current requirements of the Green Analytical Chemistry (GAC) [3] have pushed the scientific community to develop environmental-friendly strategies.

Within this context, the main efforts have been devoted to the miniaturization of sample preparation approaches, together with research on novel and greener solvents and/or sorbents [4,5]. Such miniaturization, while maintaining an adequate analytical performance, is a scientific challenge [5]. Regarding miniaturization in the SPE field (<500 mg of solid sorbent), it is important to highlight the dispersive mode, D-µSPE, which requires direct dispersion of the sorbent into the sample solution aided by agitation. D-µSPE is probably the simpler approach, as it does not require the use of additional materials such as sorbents for its performance, and it ensures a high strength of analyte-sorbent interactions.

The research on novel sorbents for µSPE and other solid-based microextraction techniques has also been a hot topic. Among outstanding materials, molecularly-imprinted polymers [6], carbonaceous nanomaterials [7], metallic nanoparticles [8], and metal-organic frameworks [9] can be mentioned.

Metal-organic frameworks (MOFs) are crystalline polymers formed by the coordination of metal ion/s or metal/s cluster with organic linkers, thus forming a highly porous and ordered three-dimensional structure [10]. Nowadays, an impressive number of MOFs has been reported, due to the infinite possible combinations between metal/cluster and linker/s. The interest awakened by MOFs in quite different fields, including analytical chemistry [11,12,13,14], is associated with their unique structural characteristics: impressive tuneability (including the possibility of post-synthetic modifications), high porosity, adequate thermal and mechanical stability, ultralow crystal densities, and the highest known surface areas.

Given these considerations, it is not surprising that MOFs have been used as novel sorbents in a variety of µSPE applications [14]. Thus, not only bare MOFs but also hybrid materials incorporating MOFs have been used in D-µSPE [12,15].

Regarding the nature of bare MOFs used in D-µSPE, MIL-101(Cr) [16,17], UiO-66 [18,19], MIL-53(Al) [20], HKUST-1 [21,22], ZIF-8 [23,24], MOF-5 [25,26], or MIL-100(Fe) [27], belonging to conventional MOFs families, have been the common choices for these studies. In the recent literature, several examples employ non-conventional MOFs [28,29].

Various strategies have been explored in the construction (election) of MOFs to achieve high sorption capacities and improved separations in µSPE. Thus, the proper choice of MOFs has been mainly performed by comparing the analytical performance under fixed extraction conditions. The main goal was to select those characteristics of MOFs that may justify efficient analyte-MOF interactions for microextraction [20,30]. In other cases, the greenness of the MOF (understood as green material itself, including its green synthesis) has been the priority aspect under consideration [31]. Most recently, the beneficial effects of MOFs, modified by the incorporation of certain functional groups in their organic linkers, in D-µSPE, has been demonstrated [18]. In this context, the “pillared-layer” strategy is considered as one of the most rational and effective ways of designing mixed-ligand frameworks [32,33]. The mixed-ligand synthetic strategy includes the advantages of different types of ligands for the rational design and construction of the desired MOFs with promising properties and potential applications [34]. Pillared-layer MOFs are promising because the structural layer motif is not generally affected by the flexibility of the pillars, and they offer different pore size/shape environments. Furthermore, the introduction of functionalities in either type of organic ligands results simple.

Among the existing pillared-layer frameworks, the Zn-triazolate-carboxylate framework [Zn_2_(tz)_2_(bdc)] (CIM-81) has quite interesting features, being constructed by a terephthalic carboxylate ligand (H_2_bdc), which bridges the 2D layers formed by Zn(II) metal ions and 1,2,4-triazole (Htz) motifs. This MOF has been previously isolated and characterized [35,36], demonstrating remarkable gas uptake capacities and adequate stability, but its performance has not been studied in any analytical applications up to date. These promising physicochemical characteristics encouraged us to explore its behavior as a novel sorbent in D-µSPE, which can open a strategy for choosing these types of structures for MOFs as sorbents in µSPE. In this way, different ligands can be included in MOFs with the purpose of expanding the interactions of the sorbent with analytes of different natures present in the environmental sample, resulting in a multi-component determination. CIM-81 is evaluated in D-µSPE combined with ultra-high-performance liquid chromatography (UHPLC), along with UV detection, for the analytical determination of nine personal care products (PCPs) – as important emerging contaminants of water samples – belonging to different PCP families, such as preservatives, UV-filters, and disinfectants.

## 2. Results and Discussion

### 2.1. Characterization of the CIM-81 MOF

CIM-81 is obtained through a solvothermal synthesis using DMA as a solvent. The synthesis could be scaled up 15-fold without loss of crystallinity or porosity. This feature is interesting for future applications of the material if intended for a large production. The MOF exhibits good thermal and chemical stability in water and in organic solvents [35,36], and it is obtained in a good yield. The chemical composition of the activated CIM-81 was confirmed by an elemental analysis, as summarized in Section 3.4.1. The nature of the as-synthesized material was confirmed by a comparison of the experimental X-ray diffraction pattern with the theoretical results already reported, as shown in Figure S1 of the ESM [35]. The structure and crystallinity of the sample after the activation procedure was also confirmed by powder X-ray diffraction (Figure S1 of the ESM). As described by Zhai’s research group [35], CIM-81 crystallizes in the space group *P4/ncc,* and each Zn^II^ center is a 4-coordinate to a carboxylate oxygen atom from the benzene-1,4-dicarboxylic ligand and to three nitrogen atoms from three different triazolate ligands. Each tz^-^ ligand binds to three Zn ions through typical μ-1,2,4˗bridging fashion, which leads to corrugated [Zn_2_(tz)_2_]_n_ two-dimensional Zn-triazolate layers. These 2D layer motifs are further connected by linear dicarboxylate pillars, thus generating the porous 3D pillared-layer structure with accessible pores of 10.8 × 6.5 Å^2^ window size (see Figure S2 of the ESM).

Figure S3 of the ESM shows the infrared spectrum before and after the solvent-exchange procedure for CIM-81. The data show the characteristic peaks of the aromatic rings and carboxyl groups, with the peak at 1663 cm^−1^ attributed to the DMA guest solvent molecules. The absence of this band in the activated crystals of CIM-81 clearly shows the absence of DMA molecules in the final framework. The new band at 1703 cm^−1^ in the activated MOF is attributed to the presence of the volatile acetone solvent in the framework supporting the solvent exchange and the activation procedure.

**Figure 1:**
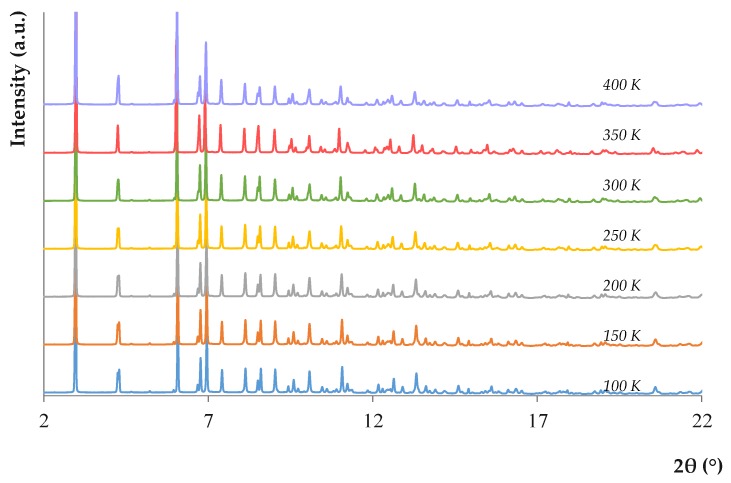
PXRD patterns of CIM-81 at different temperatures.

The study of the thermal stability was carried out by a thermogravimetric analysis and by in situ variable temperature PXRD measurements. As shown in Figure S4 of the ESM, the CIM-81 MOF shows a first weight loss below 280 °C (attributed to the elimination of the DMA solvent), and another weight loss at a higher temperature (> 400 °C), associated with the full degradation of the MOF. Results of the in situ variable temperature PXRD measurements, included in Figure 1, indicate that the framework is stable from 100 to 400 K without noticeable changes in the cell parameters. Owing to its microporous nature, the N_2_ adsorption (see Figure S5 of the ESM) at 77 K yielded a type I isotherm, with a Brunauer-Emmett-Teller (BET) surface area of 482 m^2^·g^−1^.

### 2.2. Chromatographic Method

The optimum chromatographic separation of the 9 PCPs was achieved under the conditions described in Section 3.4.2., obtaining an adequate resolution of the chromatographic peaks in 11 min. The obtained chromatographic retention times for each PCP, with the corresponding standard deviation (*n* = 60), is showed in Table S1 of the ESM, together with several quality analytical parameters of the UHPLC-UV method. The calibration curves were obtained by the injection of standard solutions with concentrations ranging from 0.01 to 1.00 µg·mL^−1^ for preservatives, and from 0.10 to 1.00 µg·mL^−1^ for the remaining PCPs. The limits of detection (LODs) and limits of quantification (LOQs) were estimated as the concentration that provided a chromatographic signal equal to 3 and 10 times the noise, respectively; subsequently, they were experimentally confirmed by the injection of standards at those levels. The intra- and inter-day precision of the UHPLC-UV method was evaluated in terms of relative standard deviations (RSD, in %), by injecting standard solutions at two levels of concentration: 0.15 and 0.45 µg·mL^−1^, *n* = 3 within the same day, and *n* = 9 over 3 non-consecutive days. For the lower concentration level, inter-day RSD values lower than 10% were obtained; these values were even lower than 6.7% for the higher level.

### 2.3. Optimization of the D-µSPE Procedure

**Figure 2:**
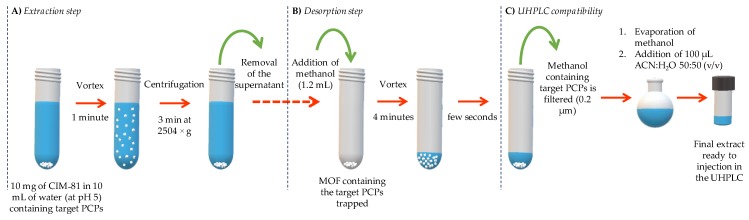
Scheme of the optimized D-µSPE-UHPLC-UV procedure using the CIM-81 MOF.

The most relevant variables for the D-µSPE-UHPLC-UV method were considered in the optimization, such as the amount of MOF sorbent, nature and volume of the desorption solvent, vortex time in the extraction step, and vortex time in the desorption step. Taking into account the importance of the desorption step when using MOFs in µSPE and D-µSPE [18,20,21], the desorption solvent was studied firstly in the optimization, and once fixed, the remaining variables were evaluated using an experimental design analysis. The scheme of the D-µSPE method is included in Figure 2, to facilitate the understanding of the variables that were considered for this optimization.

#### 2.3.1. Preliminary Studies in the D-µSPE-UHPLC-UV Method

**Figure 3:**
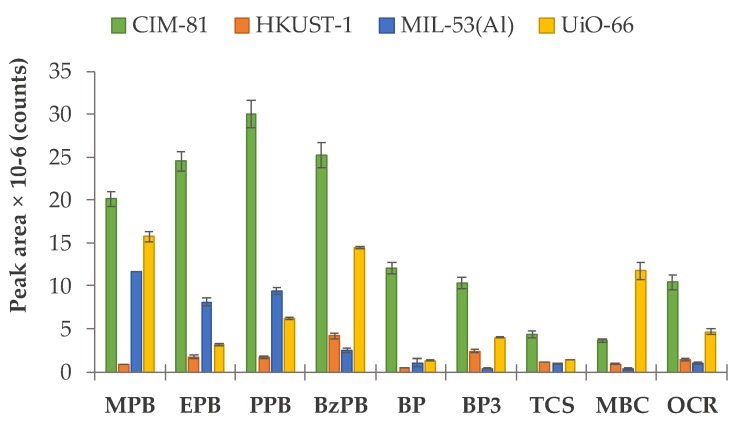
Evaluation of the extraction efficiency of PCPs using D-µSPE-UHPLC-UV and different MOFs as sorbents: CIM-81, HKUST-1, MIL-53(Al), and UiO-66; all used under the same extraction/desorption conditions (see text).

To ensure that the CIM-81 MOF could be a valid and adequate MOF to be used in the microextraction of PCPs, its performance was initially compared with that of other well-known MOFs: HKUST-1, MIL-53(Al), and UiO-66, widely used for their already demonstrated performance in analytical sample preparations [11,12,14]. Their syntheses are described in Scheme S1 of the ESM. The comparison was carried out using the same extraction conditions in all cases, taken as the initial values for the microextraction optimization: 10 mL of an aqueous standard containing 0.1 µg·mL^−1^ of PCPs at pH 5, 20 mg of the MOF, and 3 min of vortex agitation during the extraction step. Following this, the sample was centrifuged for 3 min at 2504 × g, and the supernatant was removed. PCPs were desorbed from the sorbent using 500 µL of acetonitrile, assisting the desorption by vortex agitation for 3 min. The solvent was taken, evaporated at dryness using a rotavap, and then reconstituted with 100 µL of an ACN:H_2_O mixture (ratio 50:50, *v*/*v*). The experiments were performed in triplicate. The obtained results are shown in Figure 3. It can be observed that the peak areas obtained for the majority of PCPs when using CIM-81 as the sorbent are higher than those obtained with other conventional MOFs, thus supporting the interest of using this MOF for microextraction. Given the complexity of the interactions being established, it is difficult to extract conclusions from these results, but for CIM-81 there is in the majority of the cases most likely a correlation between the size of the analyte and the extraction performance.

As mentioned above, when using solvents, the desorption conditions for MOFs in the microextraction are normally critical. Therefore, the proper selection of the desorption solvents was assessed as the first step in the optimization. In view of the polarities of the analytes, acetonitrile, acetone, trichloromethane, and methanol were the organic solvents considered for the desorption step for CIM-81 already containing trapped PCPs. Figure S6 of the ESM shows the results of this study, which was carried out under the same conditions as those used when comparing MOFs of different nature. Acetone and methanol were the solvents that were able to provide higher desorption efficiencies. The extraction efficiencies resulting from the use of methanol are adequate for the majority of PCPs, and its handling is simpler than that of acetone. For this reason, methanol was selected as a desorption solvent in the current D-µSPE-UHPLC-UV.

#### 2.3.2. Screening Optimization Study for the D-µSPE-UHPLC-UV Method

Once the MOF nature and desorption solvent nature are fixed, the remaining parameters in the optimization of the D-µSPE approach were: the amount of MOF, volume of desorption solvent (volume of methanol), and vortex time in the extraction step and in the desorption step (named, respectively, extraction time and desorption time from here on); these were subjected to a screening optimization. This screening study included sixteen random experiments (2*^n^* experiments, *n* being the number of variables). The lowest and the highest values of the variables were the following: 10 and 30 mg of CIM-81 for the amount of MOF (to ensure greenness by minimizing the amounts required), 200 and 1000 µL of methanol volume (to avoid losses of preconcentration), and 1 and 5 min for both extraction and desorption times (to have a fast procedure).

**Figure 4:**
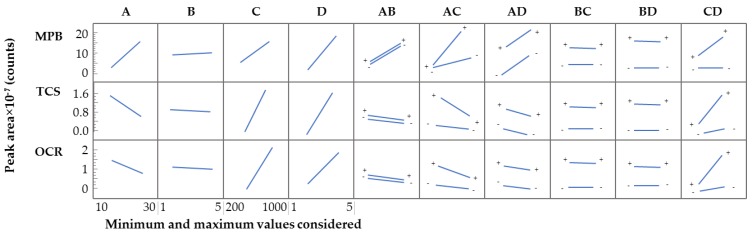
Effects of the main factors in the response (as the chromatographic peak area) for representative PCPs (MPB, TCS, and OCR), and the interaction among factors obtained in the screening analysis of the main variables that influence the D-µSPE-UHPLC-UV; where **A** is the amount of MOF, **B** is the extraction time, **C** is the volume of methanol, and **D** is the desorption time.

Figure 4 shows the obtained results of the effects of the main factors, and the interaction among the factors, for representative PCPs (covering examples of the three families: preservatives, UV-filters and disinfectants). The screening study showed that, for all PCPs, the extraction time has no influence, while the volume of methanol and the desorption time present a positive influence in the entire extraction efficiency. On the other hand, only for MPB, EPB, and PPB, the extraction efficiencies are improved significantly when the amount of sorbent CIM-81 increased. Based on these screening results, the extraction time was fixed to the minimum value (1 min). Regarding the amount of CIM-81, given its low influence for the majority of PCPs, it was fixed to 10 mg to ensure greenness and low costs.

#### 2.3.3. Doehlert Design for the D-µSPE-UHPLC-UV Method

The most influential variables (volume of methanol and desorption time) dictated by the screening analysis, were evaluated with a Doehlert experimental design of two factors. In this design, the experimental points are located in a circle of radius 1, with a spatial distribution located in the vertices of a hexagon [37]. The total number of experiments (*n*) is given by the following equation:

*n* = *k^2^* + *k* + 1 (1)

where *k* is the number of factors. The adequate assignation of the Doehlert codes according to the relative influence of the variables considered was also important, because the codes for factors 1 and 2 implied 5 and 3 levels, respectively. Taking into account that the volume of the desorption solvent is related to the amount of CIM˗81, and that this value was fixed, the desorption solvent volume requires less attention than the desorption time. Therefore, the desorption time was selected as factor 1, and the volume of methanol as factor 2. In this case, the ranges studied were between 1 and 7 min for the desorption time, and between 0.2 and 2.0 mL for the volume of methanol. The relationship between the coded and real values is given by the following equation:

(2) $$C_{i}=\frac{\left(X_{i}-X_{i}^{0}\right)}{\Delta X_{i}}\alpha$$

where *C_i_* is the coded value for the level of factor *i*, *X_i_* is its real value in an experiment, *X^0^_i_* is the real value at the center of the experimental domain, Δ*X_i_* is the variation step of the real value, and α is the coded value limit for each factor. The code values of a Doehlert design of two factors (implying seven experiments) and the real values of each variable studied are detailed in Table S2 of the ESM.

A regression equation was used (Equation (3)) for the correlation between the response of each experiment and the factors:

*R* = constant + *A*[*t_desorption_*] + *B*[*mL_methanol_*] + *AA*[*t_desorption_*]^2^ + *AB*[*t_desorptionn_*][*mL_methanol_*] + *BB*[*mL_methanol_*]^2^ (3)

**Figure 5:**
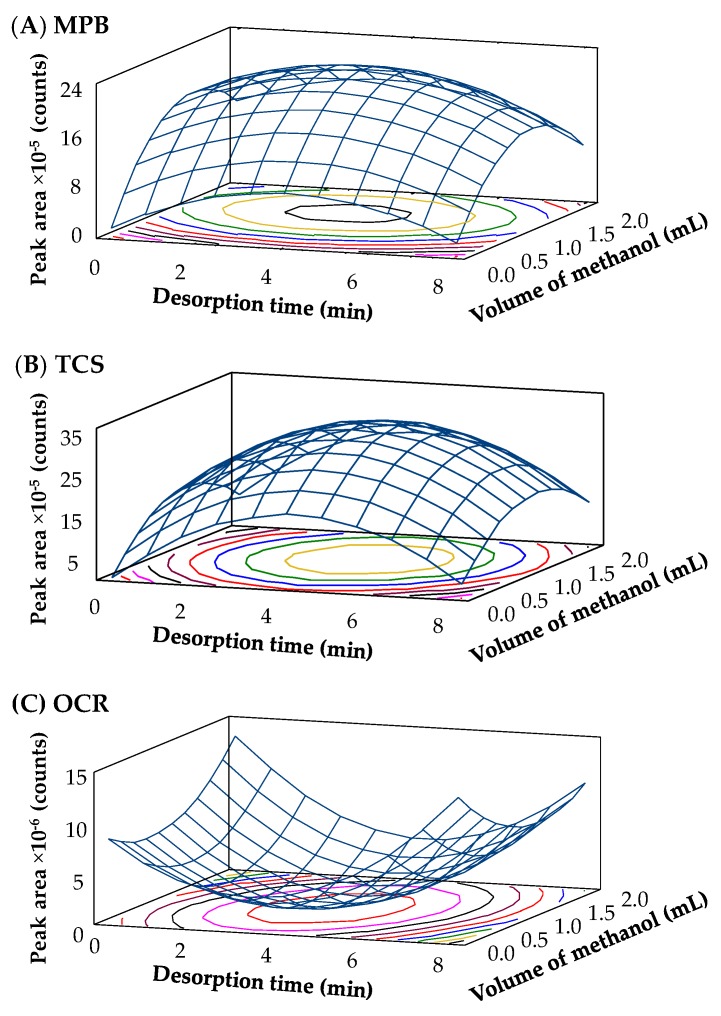
Surface responses obtained for representative PCPs: (**A**) MPB, (**B**) TCS, and (**C**) OCR, obtained through the statistical study of the Doehlert experimental design.

The constants and Doehlert coefficients for each PCP are included in Table S3 of the ESM. A response surface methodology permitted the selection of the optimum conditions of the variables for each PCP, which are also included in Table S3 of the ESM. Figure 5 shows the three-dimensional response surfaces obtained (desorption time × volume of methanol × peak areas). In general, the optimum value for the desorption time is around 4 min, except for PPB, BzPB, and BP (7 min), and for OCR (1 min). Regarding the volume of the desorption solvent, an adequate desorption of the PCP is reached using 1.2 mL of methanol, except for BP and OCR (2.0 mL). According to these results, 4 min of desorption time and 1.2 mL of methanol were selected as compromise optimum conditions for the group of PCPs studied.

It is also possible to calculate the variation between the response obtained with the particular optimum conditions for each PCP *versus* the compromise conditions selected, using Equation (3) and the coefficients showed in Table S3 of the ESM. The calculated variation was lower than 30% for all PCPs, except for BP and OCR. These exceptions are linked to the fact that their optimum conditions are quite near to the limits imposed on the variables studied during the optimization. Thus, the optimum conditions obtained in the Doehlert design (4 min of desorption time and 1.2 mL of methanol) are adequate for the target PCPs.

### 2.4. Analytical Performance of the Optimized D-µSPE-UHPLC-UV Method

The analytical performance of the D-µSPE-UHPLC-UV method was evaluated by obtaining several quality analytical parameters. The extraction efficiency and the intermediate precision was carried out at two concentration levels, with the low and intermediate values taking into account the working range of the calibration curve. The obtained results are summarized in Table 1.

The working range of the calibration curves, performed for the entire method, was from 5 to 100 µg·L^−1^, with a determination coefficient (R^2^) higher than 0.995 for the 7 calibration levels that were used. The sensitivity of the method, expressed as the slope of the calibration curve, ranged from (36 ± 1) × 10^−4^ for TCS, to (350 ± 1) × 10^−4^ for OCR. LODs and LOQs were estimated as the concentration that provided a chromatographic signal equal to 3 and 10 time the noise, respectively, and then experimentally confirmed by the preparation of aqueous standards at those levels. LODs ranged between 0.5 µg·L^−1^ for OCR and 1.5 µg·L^−1^ for BP, and LOQs ranged between 1.7 µg·L^−1^ for OCR and 5.0 µg·L^−1^ for BP.

The intermediate precision of the method was evaluated in terms of RSD (in %) for 3 experiments carried out in the same day (intra-day) and for 9 experiments in three different non-consecutive days (inter-day). For the lowest concentration level tested (15 µg·L^−1^), the intra-day RSD value was lower than 8.7% for BP, while the inter-day RSD value was lower than 13% for BP3.

The extraction performance was evaluated in terms of relative recoveries (RR) and enrichment factors (E_F_), using aqueous standards at the same concentration levels. The average RR values were 115% and 100% for 15 µg·L^−1^ and 45 µg·L^−1^, respectively. The E_F_ values ranged from 3.6 for MBC to 34 for OCR at 15 µg·L^−1^, and from 2.6 for BP to 29 for OCR at 45 µg·L^−1^.

### 2.5. Reuse of the CIM-81 MOF in the D-µSPE-UHPLC-UV Method

Within a green analytical chemistry perspective, a study of the reuse of CIM-81 as a sorbent was carried out. Thus, the MOF used in previous extraction experiments was thoroughly cleaned, and its analytical performance was evaluated in terms of recovery and precision. To accomplish this, the CIM-81 MOF was washed three times with 1 mL of methanol (desorption solvent), and later subjected to the activation procedure described in Section 3.4.1. Following this, the MOF was used as a sorbent in the optimized D-µSPE-UHPLC-UV method. The reuse study, evaluated by the precision and the extraction performance of the entire method, was carried out using two aqueous standards: 15 and 45 µg·L^−1^. The obtained results are summarized in Table S4 of the ESM.

For both concentration levels, the RSD (*n* = 3) ranged between ~4% and ~15%, confirming the high precision of the method, this time reusing the CIM-81 a second time. Regarding the relative recovery values, they ranged from 82.9% for OCR to 129% for TCS at the lower concentration level, and from 74.9% for EPB to 102% for MPB at the intermediate concentration level. An analysis of variance (ANOVA) was performed to determine whether there were significant differences in the average RR values obtained in the validated method and in the reuse study. The ANOVA study indicated that there were no such differences among the results obtained (α = 0.05). Furthermore, the obtained E_F_ values are practically the same for all PCPs as they were before (see Table 1 for a better comparison of the results).

The structure and crystallinity of the extractant material was assessed after each cycle by means of a powder X-ray diffraction pattern (see Figure S7 of the ESM). After the first extraction cycle, some roughening of the diffraction peaks is observed, but the crystal structure is kept. However, after the second extraction cycle there is a pronounce roughening of the diffraction peaks, but the main ones corresponding to the original structure are still present, indicating some degradation of the framework that possibly compromises the pore structure of the CIM-81 material. Therefore, the CIM-81 MOF can be used twice satisfactorily. A third reuse is not possible due to heavy alterations of the CIM-81 pore structure.

### 2.6. Analysis of Samples

Once the entire D-µSPE-UHPLC-UV method was optimized and validated, a total of three wastewater samples were analyzed, named Sample 1, Sample 2, and Sample 3. As summarized in Table 2, 4 out of the 9 PCPs studied (MPB, EPB, TCS, and MBC) were quantified in Sample 1, while 3 of them were quantified in Sample 2 (MPB, TCS, and OCR). MPB and TCS were quantified both in Sample 1 and Sample 2 at concentrations of 7.4 ± 0.8 µg·L^−1^ for MPB and 15 ± 1 µg·L^−1^ for TCS, and 7.6 ± 0.9 µg·L^−1^ for MPB and 28 ± 2µg·L^−1^ for TCS, respectively. Their high and intense use explain the presence of these contaminants in these kinds of samples. Other PCPs that were determined were EPB (13.2 ± 0.3 µg·L^−1^) and MBC (16 ± 1 µg·L^−1^) in Sample 1, and OCR (0.6 ± 0.1 µg·L^−1^) in Sample 2. For the remaining samples, PCPs were not detected.

**Figure 6:**
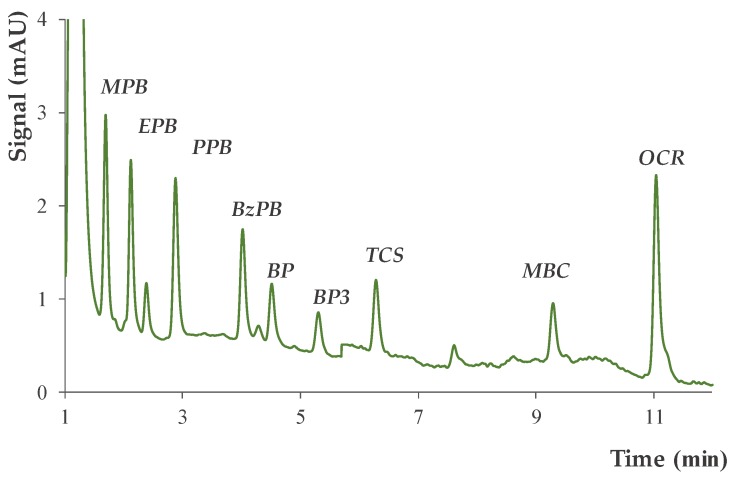
Representative chromatogram obtained by subjecting a wastewater sample (spiked at 45 µg·L^−1^) to the overall optimized D-µSPE-UHPLC-UV method.

Possible matrix effects were evaluated in Sample 3 (in which no PCPs were detected), by spiking it at two levels (15 and 45 µg·L^−1^). Table 2 shows the resulting relative recoveries and enrichment factors. The average relative recoveries were 112 and 107%, with enrichment factors ranging from 2.8 to 29 and from 2.6 to 24, at the lower and higher spiked levels, respectively. These values clearly agree with those obtained with aqueous standards, thus showing the absence of matrix effects. Figure 6 shows a representative chromatogram, obtained in the analysis of Sample 3 (spiked at 45 µg·L^−1^).

## 3. Experimental Section

### 3.1. Standards, Reagents and Materials

Nine PCPs were considered in this study: four preservatives, four UV-filters, and one disinfectant. Methylparaben (MPB), ethylparaben (EPB), propylparaben (PPB), and triclosan (TCS) were purchased from Dr Ehrenstorfer GmbH (Augsburg, Germany); meanwhile, benzylparaben (BzPB), benzophenone (BP), benzophenone-3 (BP3), 3-(4-methylbenzylidene) camphor (MBC), and octocrylene (OCR) were supplied by Sigma-Aldrich (Steinheim, Germany). All standards presented a purity higher than 98%. Individual stock solutions were prepared in acetonitrile, at concentrations between 1000 and 4000 µg·mL^−1^. Intermediate standard solutions were prepared weekly by the dilution of the stock solutions using acetonitrile as a solvent, mixing all PCPs in one single standard solution. Working aqueous standard solutions were also prepared daily. All solutions were stored at 4 °C, protected from the light.

Ultrapure deionized water was obtained using the Milli-Q gradient A10 water purification system from Millipore (Watford, UK). Acetonitrile (ACN) of LC-MS grade was purchased from VWR International (Barcelona, Spain), methanol of LC-MS grade from Honeywell (Seelze, Germany), acetone and trichloromethane for HPLC from Sigma-Aldrich. An acetic/acetate buffer solution was prepared, using glacial acetic acid acquired from Merck (Darmstadt, Germany), and anhydrous sodium acetate (>99%) from Sigma-Aldrich.

The reagents 1,2,4-triazol (Htz) and benzene-1,4-dicaboxilic acid (H_2_bdc) were used for the synthesis of the CIM-81 MOF, with a purity of 98%; they were supplied by Sigma-Aldrich, whereas the zinc nitrate hexahydrate (98%) was acquired from Honeywell. *N,N*-dimethylacetamide (DMA) was purchased from Merck. The 45 mL teflon solvothermal reactors and the stainless-steel autoclaves for the synthesis of MOF were supplied by Parr Instrument Company (Moline, IL, USA).

Pyrex^®^ centrifuge tubes (Staffordshire, UK), a glass syringe Fortuna Optima^®^ of 2 mL capacity (Sigma-Aldrich), and 0.2 μm PVDF (polyvinylidene fluoride) Whatman™ syringe filters (GE Healthcare, Buckinghamshire, UK) were all used for the microextraction procedures.

### 3.2. Sample Collection

Three wastewater samples were analyzed in this study. Wastewaters were provided by an environmental analysis laboratory from Tenerife (Canary Islands), after sampling the effluents of different wastewater treatment plants. The sampling was carried out in bottles of 200 mL, ensuring the absence of air bubbles. The samples were kept in a portable fridge until they reached the laboratory, before being stored at 4 °C and protected from light until the analysis (no more than one week in the fridge).

### 3.3. Instrumentation

A chromatographic analysis was carried out using a UHPLC 1260 Infinity Series from Agilent Technologies. The equipment has a quaternary pump, an InfinityLab Poroshell 120 EC-C18 column (4.6 × 50 mm × 2.7 µm), supplied by Agilent Technologies, and a Rheodyne 7725i injection valve with a loop of 5 µL. The detector used was a Vis-UV ProStar 325 LC Detector Series from Varian (Palo Alto, CA, USA).

A Reax Top vortex from Heidolph (Schwabach, Germany), a centrifuge from a Eppendorf^®^ model 5720 (Hamburg, Germany), and the rotary evaporator IKA RV10 with a vacuum controller CVR 3000 supplied by VWR, were all used in the extraction procedure. The oven used for the synthesis of the CIM-81 MOF was the Universal UF30 model supplied by Memmert (Schwabach, Germany).

The phase identification of the MOF crystals was carried out by X-ray powder diffraction. The X’Pert Diffractometer supplied by PANalytical (Eindhoven, Netherlands) operating with Bragg-Brentano geometry was used. The data collection was carried out using Cu K1 radiation (λ = 1.5418 Å) over the angular range of 5.01° to 80.00° (0.02° steps), with a total exposure time of 30 min. High resolution powder diffraction patterns at different temperatures were measured at the BL04-MSPD beamline [38] of the ALBA synchrotron (Barcelona, Spain) at 17.5 keV (λ = 0.70815 Å), equipped with the Mythen-II detector (Dectris) in the 0.37–43.2°angular range.

IR spectra (450–4000 cm^−1^) were recorded for the powdered crystals by an IRAffinity1 spectrophotometer from Shimadzu (Kyoto, Japan) equipped with a Pike technologies GladiATR.

The nitrogen adsorption isotherms were measured on the surface area analyzer Gemini V 2365 Model, supplied by Micromeritics (Norcross, GA, USA), at 77 K in the range of 0.02 ≤ P/P_0_ ≤ 1.00. The Brunauer, Emmet and Teller (BET) method was used to calculate the surface area.

A thermogravimetric analysis on freshly crushed crystals of CIM-81 was carried out in a thermal analyzer Perkin-Elmer Pyris Diamond TG/DTA (Waltham, MA, USA), typically requiring a few milligrams placed on an alumina crucible under a nitrogen atmosphere at a flow rate of 20 cm^3^·min^−1^. The temperature was ramped from 25 to 250 °C at a heating rate of 5 °C min^−1^.

Elemental analyses (C, H, N) were carried out with an elemental analyzer CNHS Flash EA 1112 from Thermo Fisher Scientific (Massachusetts, MA, USA).

The Statgraphics Centurion XV version 15.1.02 software was used for the statistical analyses (Statgraphics Technologies, The Plains, VA, USA).

### 3.4. Procedures

#### 3.4.1. Synthesis of MOF

The CIM-81 MOF, formulated as [Zn_2_(tz)_2_(bdc)]·2DMA, was synthetized through a solvothermal method. A mixture of Zn(NO_3_)_2_·6H_2_O (592 mg, 2 mmol), 1,2,4-triazole (140 mg, 2 mmol), and benzene-1,4-dicarboxylic acid (170 mg, 1 mmol), was dissolved in 15 mL of DMA. The final mixture was placed in a Parr Teflon-lined stainless steel vessel (25 mL) under autogenous pressure, and heated at 120 °C for 72 h. The resulting colorless crystals were filtered by gravity, washed with DMA and acetone, and dried at 50 °C. Afterwards, the MOF was immersed in acetone for 24 h to remove the nonvolatile solvates (this process was repeated two times) followed by drying at 100 °C. The activated CIM-81 was then ready to use in the analytical procedure. The obtained yield was 83%. The elemental analysis calculated for C_12_H_10_O_4_N_6_Zn_2_ (in %) was: C, 33.34; H, 1.87; N, 19.50; exp. C, 33.14, H, 1.73, N, 19.94.

#### 3.4.2. Chromatographic Method

The optimum separation required a binary mobile phase composed of acetonitrile and water with 0.1% (*v*/*v*) of acetic acid in the aqueous phase, at a constant flow rate of 0.5 mL·min^−1^. The chromatographic separation was carried out at constant temperature of 25 °C. The optimum separation was obtained by the follow gradient: initially 50% (*v*/*v*) of acetonitrile, that was maintained isocratic during 1 min, followed by a linear elution gradient from 50% to 90% (*v*/*v*) of acetonitrile up to 8 min; finally, the acetonitrile was maintained at 90% (*v*/*v*) for 4 additional minutes. The wavelength of the detector was fixed initially at 254 nm, and was changed at 5.7 min to 289 nm, being then maintained through the end of the chromatographic run.

#### 3.4.3. Dispersive Miniaturized Solid-Phase Extraction (D-µSPE) Method

The microextraction procedure was carefully optimized. The optimum method required the addition of 10 mg of the CIM-81 MOF to 10 mL of the water sample (or aqueous standard), having the pH adjusted to 5 with an acetic/acetate buffer. The aqueous sample (or aqueous standard) was then subjected to vortex stirring for 1 min and then centrifuged (2504 × g) for 3 min. The MOF was then separated to accomplish the desorption stage using both 1.2 mL of methanol and a vortex agitation of 4 min. The desorption solvent (methanol) containing the extracted and preconcentrated analytes was then filtered (0.2 µm) and subjected to dryness under vacuum evaporation, followed by a reconstitution in 100 µL of the chromatographic mobile phase corresponding to the initial conditions (a mixture of 50:50 (*v*/*v*) of ACN:H_2_O).

## 4. Conclusions

In order to prepare the terephthalate pillared Zn-triazolate metal organic-framework CIM-81, a mixed-ligand synthetic strategy was employed successfully. In this type of MOF, the flexibility of the pillars does not affect the structural layer motif, while offering different pore size/shape environments in the structure.

The as-synthetized and characterized CIM-81 was tested the first time as a sorbent via a D-µSPE method for a group of 9 personal care products in complex wastewaters, demonstrating better analytical performance features than others MOFs that had already been used with success, such as HKUST-1, MIL-53(Al), and UiO-66.

The D-µSPE method with CIM-81 as a sorbent, combined with UHPLC-UV, was adequately optimized, by first using a screening and afterwards a Doehlert design. The optimized microextraction method included a number of environmental-friendly characteristics, such as: low amounts of MOF (10 mg); low sample volumes (10 mL); short sample preparation times (8 min for extraction and desorption, and 10 min for the solvent exchange step); and the minimization of the organic solvent consumption (1.2 mL of methanol in D-µSPE and ~4 mL of acetonitrile in the chromatographic run). The developed method was sensitive, with limits of detection down to 1.5 ng·mL^−1^, and it results in an adequate analytical performance in terms of relative recovery, enrichment factor and intermediate precision, while dealing with complex samples such as wastewaters.

The promising results achieved with this pillared-layer MOF can open a new line of enquiry to tailor MOFs to microextraction strategies, particularly in view of the simplicity of functionalization of organic ligands in these types of crystalline compounds.

## Figures and Tables

**Table 1:** Several quality analytical parameters of the optimized D-µSPE-UHPLC-UV method.

PCP	(Slope ± S_b_^a^) × 10^˗4^	R^2 b^	S_y/x_^c^ × 10^−3^	LOD/LOQ ^d^ (µg·L^−1^)	E_F_^e^	Conc. Level: 15 µg·L^−1^			Conc. Level: 45 µg·L^−1^		
						*RSD*^f^ (%) intra-day/inter-day	*RR*^g^ (%)	*E_F_* ^h^	*RSD*^f^ (%) intra-day/inter-day	*RR*^g^ (%)	*E_F_* ^h^
MPB	207 ± 6	0.9964	5.1	0.7/2.3	5.3	5.1/7.2	122	9.1	2.3/7.1	108	6.6
EPB	233 ± 5	0.9973	5.0	1.0/3.3	6.8	5.0/6.5	125	7.2	2.6/5.1	111	7.1
PPB	247 ± 7	0.9963	6.2	0.8/2.7	8.1	5.5/9.4	108	8.1	5.6/8.5	97.7	7.7
BzPB	180 ± 4	0.9976	3.7	0.9/3.0	6.7	6.6/8.3	121	7.4	3.7/7.0	115	7.5
BP	121 ± 4	0.9957	3.3	1.5/5.0	3.1	8.7/10	112	3.6	4.4/9.4	80.9	2.6
BP3	139 ± 4	0.9961	3.6	1.2/4.0	5.4	6.2/13	118	6.6	6.7/9.4	97.8	5.3
TCS	36 ± 1	0.9971	0.80	1.0/3.3	5.7	7.2/11	94.1	12	4.1/8.4	92.8	7.2
MBC	116 ± 3	0.9956	3.2	1.4/4.7	3.3	4.6/6.6	120	3.6	2.6/8.2	99.5	3.2
OCR	350 ± 4	0.9992	4.0	0.5/1.7	29	6.6/12	112	34	8.0/6.0	96.2	29

^a^ Standard deviation associated with the slope; ^b^ Determination coefficient; ^c^ Error of the estimate (standard deviation of the residuals); ^d^ Limit of detection and limit of quantification, obtained as described in the text; ^e^ Enrichment factor calculated as ratio of the calibration slope of the chromatographic method and the calibration slope of the overall method; ^f^ Relative standard deviation: intra-day (*n* = 3) and inter-day (*n* = 9 in three different non-consecutive days); ^g^ Relative recovery; ^h^ Enrichment factor.

**Table 2:** Contents of PCPs (µg·L^−1^) found in the analysis of wastewater samples with the entire D-µSPE-UHPLC-UV method proposed for the monitoring of PCPs, and recovery study in these samples at two spiked levels.

PCP	Sample 1	Sample 2	Sample 3 *			
			Spiked Level: 15 µg·L^−1^		Spiked Level: 45 µg·L^−1^	
			*RR*^a^ (%)	*E_F_* ^b^	*RR*^a^ (%)	*E_F_* ^b^
MPB	7.4 ± 0.8	7.6 ± 0.9	101	8.0	113	6.9
EPB	13.2 ± 0.3	n.d.	122	7.0	119	7.6
PPB	n.d.	n.d.	120	9.1	112	8.9
BzPB	n.d.	n.d.	109	6.6	120	7.8
BP	n.d.	n.d.	128	4.2	90.1	2.9
BP3	n.d.	n.d.	112	6.2	122	6.6
TCS	15 ± 1	28 ± 2	127	14	121	8.7
MBC	16 ± 1	n.d.	97.2	2.8	82.9	2.6
OCR	n.d.	0.6 ± 0.1	95.8	29	82.2	24

^a^ Relative recovery; ^b^ Enrichment factor; n.d. non-detected; * PCPs were not detected in Sample 3.

## Supplementary Materials

The Supplementary Materials are available online.

## Abbreviations

ACN	acetonitrile
BET	Brunauer, Emmet and Teller
BP	benzophenone
BP3	benzophenone-3
BzPB	benzylparaben
DMA	*N-N*˗dimethylacetamide
D-µSPE	dispersive miniaturized solid phase extraction
EPB	ethylparaben
GAC	Green Analytical Chemistry
LLE	liquid-liquid extraction
MBC	3-(4-methylbenzylidene) camphor
MOF	metal-organic frameworks
MPB	methylparaben
OCR	octocrylene
PPB	propylparaben
PCPs	personal care products
SBSE	stir-bar sorptive microextraction
SCSE	stir-cake sorptive microextraction
SPE	solid-phase extraction
SPME	solid-phase microextraction
TCS	triclosan
µSPE	miniaturized solid phase extraction
UHPLC	ultra-high performance liquid chromatography
