# Supplementary material for: Application of a Pillared-Layer Zn-Triazolate Metal-Organic Framework in the Dispersive Miniaturized Solid-Phase Extraction of Personal Care Products from Wastewater Samples
Source: Molecules. 2019 Feb 15;24(4):690. doi: 10.3390/molecules24040690 (PMC6412874; doi:10.3390/molecules24040690)
Supplement: Supplementary file 1 [file molecules-24-00690-s001.pdf]

# Supplementary materials

## Application of a Pillared-Layer Zn-Triazolate Metal-Organic Framework in the Dispersive Miniaturized Solid-Phase Extraction of Personal Care Products from Wastewater Samples

**Providencia González-Hernández [1], Ana B. Lago [2], Jorge Pasán [2], Catalina Ruiz-Pérez [2], Juan H. Ayala [1], Ana M. Afonso [1] and Verónica Pino [1,3,*]**

[1] Departamento de Química, Unidad Departamental de Química Analítica, Universidad de La Laguna (ULL), La Laguna, Tenerife 38206, Spain
[2] Laboratorio de Rayos X y Materiales Moleculares (MATMOL), Departamento de Física, Universidad de La Laguna (ULL), La Laguna, Tenerife 38206, Spain
[3] University Institute of Tropical Diseases and Public Health, Universidad de La Laguna (ULL), La Laguna, Tenerife 38206, Spain

**Table of contents**

Figure S1 ........ page S1

Figure S2 ........ page S2

Figure S3 ........ page S3

Figure S4 ........ page S4

Figure S5 ........ page S5

Figure S6 ........ page S6

----------------------------------------------------------------------------------------------------

Scheme ........ page S7

----------------------------------------------------------------------------------------------------

Table S1 ........ page S8

Table S2 ........ page S9

Table S3 ........ page S10

Table S4 ........ page S11

**Figure S1**. XRD patterns of (**A**) activated CIM-81, (**B**) CIM-81 without activation, and (**C**) theoretical XRD pattern.

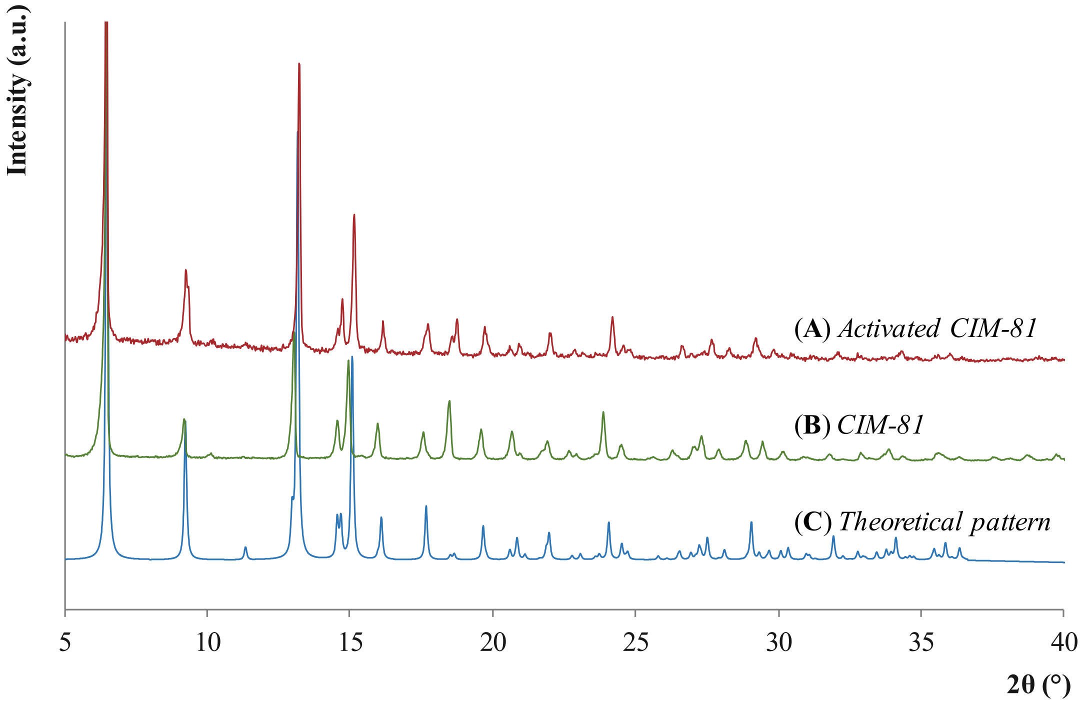

**Figure S2.** A view of the crystal structure of CIM-81, obtained of ref. (36), along the crystallographic a (left) and c axes (right).

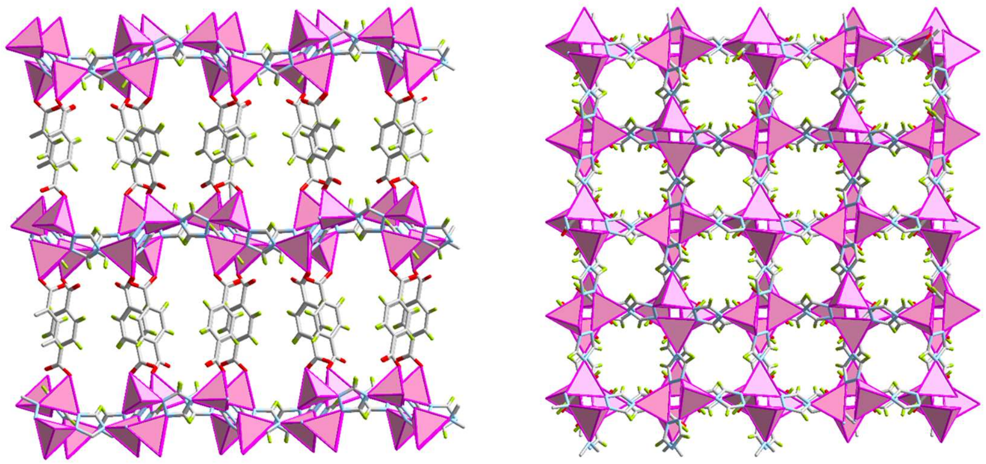

**Figure S3**. FT-IR spectra of CIM-81 before the solvent-exchange procedure (red line), and after the activation procedure (green line).

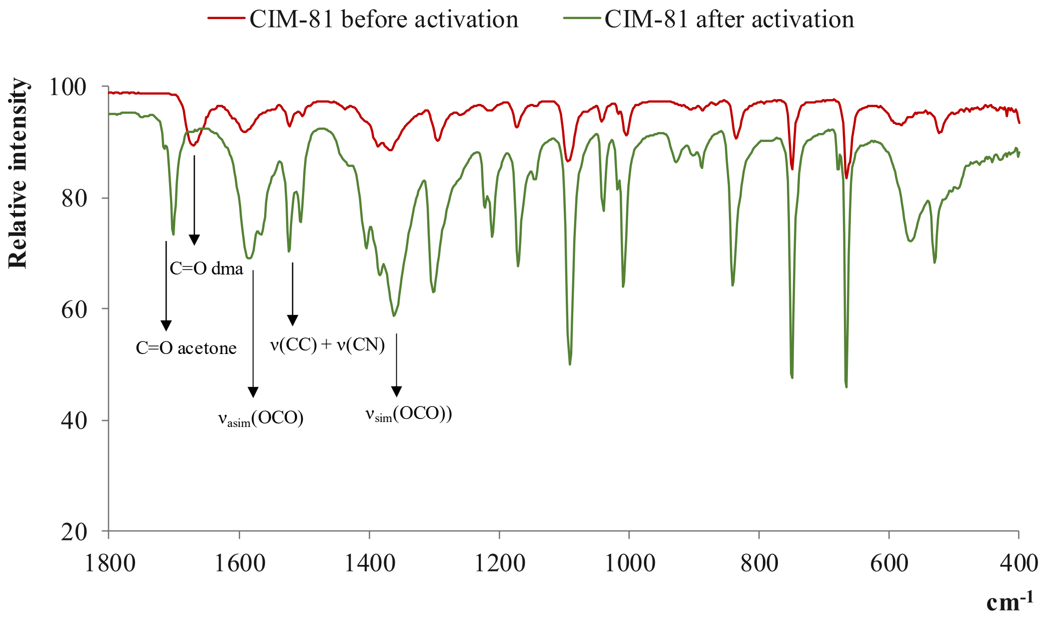

**Figure S4**. Thermogravimetric curve of CIM-81.

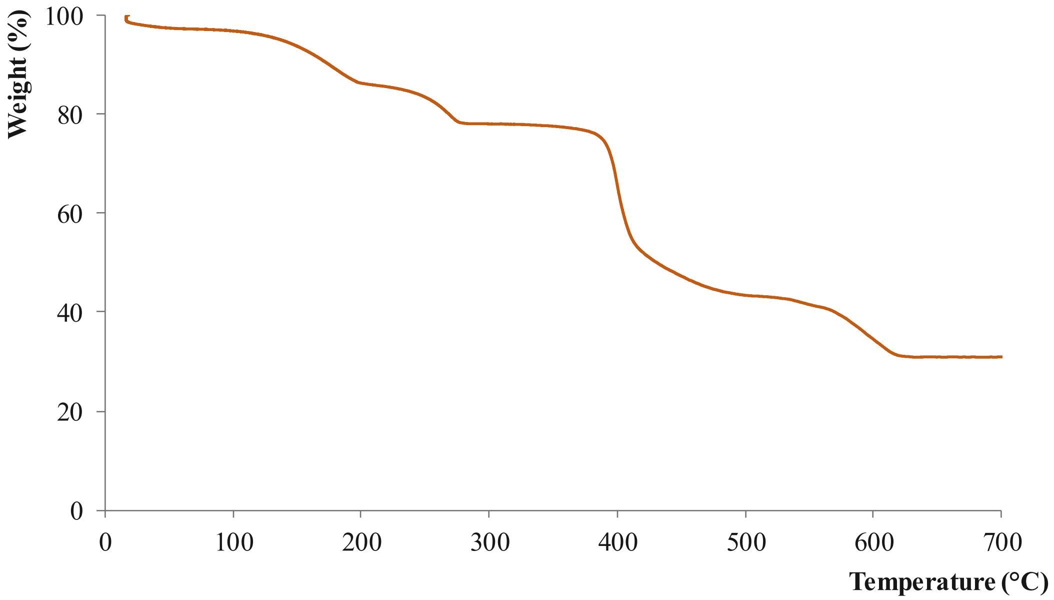

**Figure S5**. Nitrogen adsorption isotherm of CIM-81 at 77K.

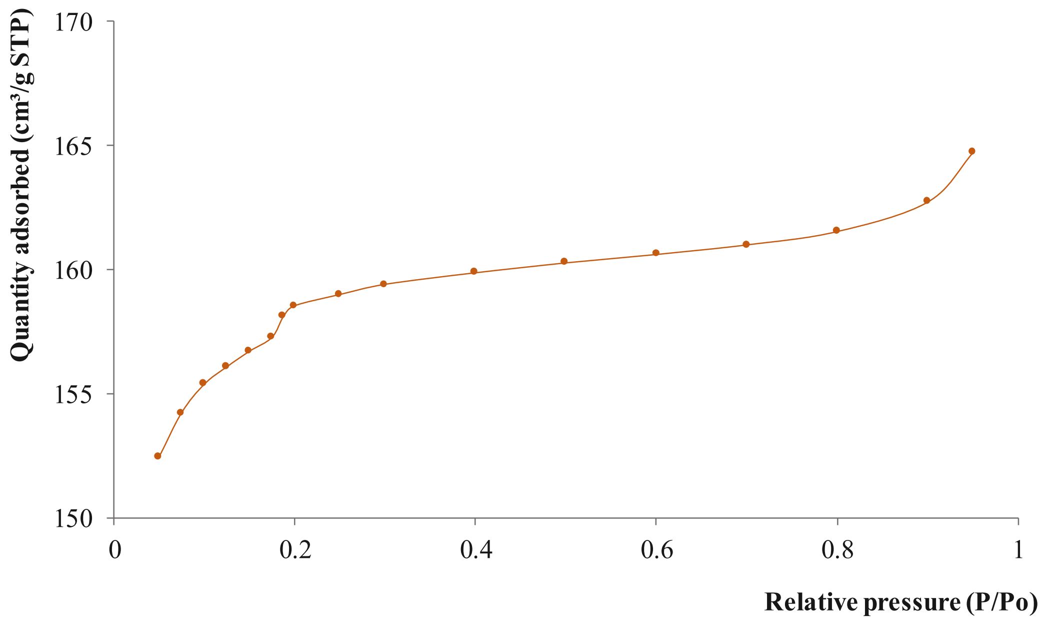

**Figure S6**. Evaluation of several organic solvents as possible desorption solvents for PCPs trapped by the MOF CIM-81 in D-µSPE-UHPLC-UV. Specific conditions of the experiments described in the text.

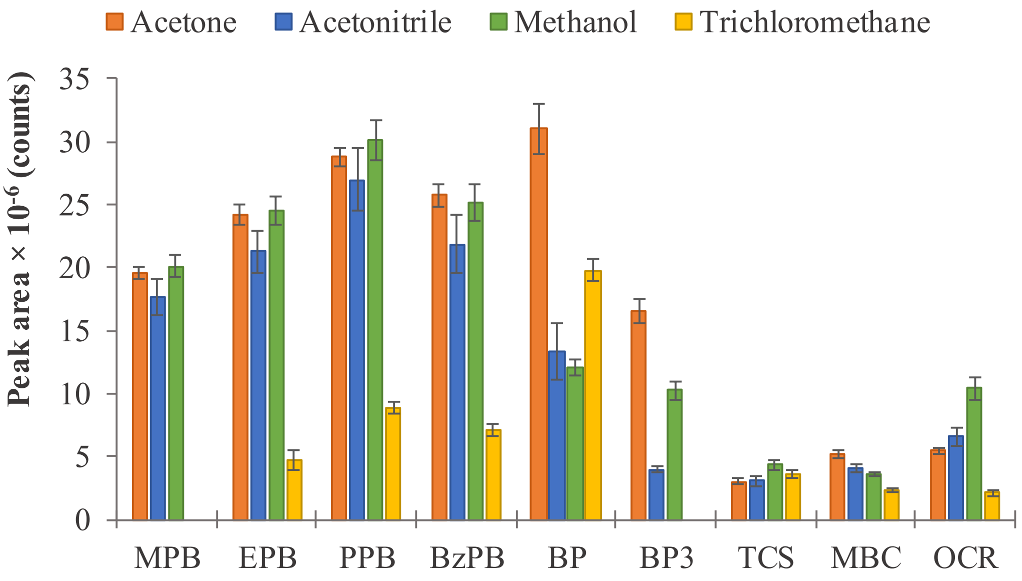

**Figure S7**. XRD patterns of (**A**) activated CIM-81, (**B**) CIM-81 after one extraction cycles, and (**C**) CIM-81 after two extraction cycles.

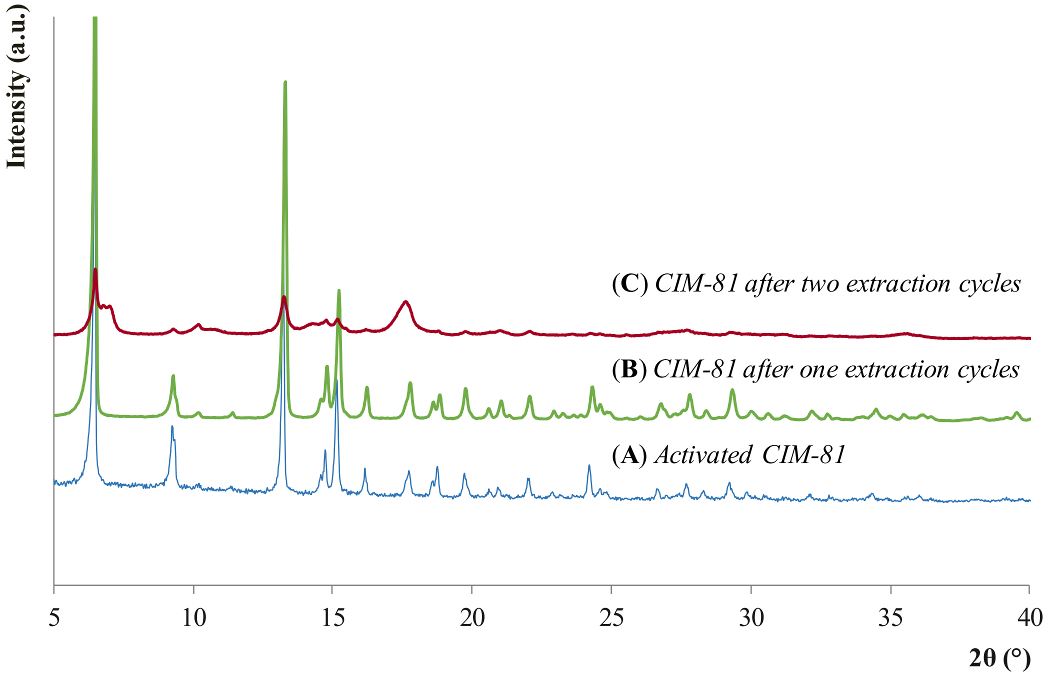

**Scheme**

*Reagents to synthetize MOFs*

Copper(II) nitrate hemipentahydrate (98%), zirconium chloride (98%), aluminum(III) nitrate nonahydrate (98%), benzene-1,3,5-tricarboxylic acid (95%) ($H_3btc$), and benzene-1,4-dicarboxilic acid ($H_2bdc$) (98%), were purchased from Sigma-Aldrich. Absolute ethanol and methanol were supplied by Panreac (Barcelona, Spain). Dimethylformamide was acquired to Merck KGaA (Darmstadt, Germany) and hydrochloric acid (37%) to Honeywell (Seelze, Germany).

*Synthesis of MOFs*

All MOFs were synthetized by a solvothermal method.

HKUST-1, formulated as $[Cu_3(btc)_2(H_2O)_3]$, was synthetized following our previous study [22]. An aqueous solution of $Cu(NO_3)_2 \cdot 5H_2O$ (1.255 g, 5.4 mmol) was added dropwise to 15 mL of an ethanol solution of $H_3btc$ (0.630 g, 3 mmol), followed by stirring for 20 min. The mixture was transferred to the solvothermal reactor (45 mL), and then heated to 110 ºC during 24 hours. The obtained blue crystals were filtered by gravity, washed with ethanol, and dried at room temperature. HKUST-1 was heated at 150 ºC for 24 hours in order to remove guest molecules from the pores of the crystalline structure. The yield was 90%.

MIL-53(Al), formulated as [AlOH(bdc)], was synthetized following our previous study [22]. $Al(NO_3) \cdot 9H_2O$ (1300 mg, 3.5 mmol) and benzene-1,4-dicarboxylic acid (288 mg, 1.7 mmol) were dissolved in 15 mL of ultrapure water. The mixture was transferred

to the solvothermal reactor (23 mL), and heated to 220 ºC during 72 hours. The obtained crystals were filtered by gravity, washed with ultrapure water, and dried at 50 ºC for 24 hours. MIL-53(Al) was heated at 400 ºC for 16 hours in order to remove guest molecules from the pores of the crystalline structure. The yield was 45%.

UiO-66, formulated as [$Zr_6O_4(OH)_4(bdc)_6$], was synthetized following our previous study [19]. $ZrCl_4$ (233 mg, 1 mmol) and benzene-1,4-dicarboxylic acid (246 mg, 1.5 mmol) was dissolved in 15 mL of dimethylformamide and 1 mL of concentrated HCl. The mixture was transferred to the solvothermal reactor (45 mL), and heated to 150 ºC during 24 hours. The obtained colorless crystals were filtered by gravity, and washed with dimethylformamide and methanol. UiO-66 was then heated at 150 ºC for 24 hours in order to remove guest molecules from the pores of the crystalline structure. The yield was 95%.

*Characterization of MOFs*

All MOFs were characterized by XRD, IR and $N_2$ adsorption measurements, showing that the synthesized compounds are in agreement with the previously reported data.

**Table S1**. Several quality analytical parameters of the UHPLC-UV method.

| PCP | $t_R \pm$ SD (n = 60) | Working range (µg·mL$^{-1}$) | (Slope ± $S_b$[a]) × $10^{-4}$ | $R^2$ [b] | $S_{y/x}$[c] × $10^{-3}$ | LOD[d] (µg·L$^{-1}$) | LOQ[e] (µg·L$^{-1}$) | *RSD*[f] *(%) intra-day / inter-day* (0.15 µg·mL$^{-1}$) | *RSD*[f] *(%) intra-day / inter-day* (0.45 µg·mL$^{-1}$) |
|---|---|---|---|---|---|---|---|---|---|
| MPB | 1.68 ± 0.03 | 0.01 – 1.00 | 390 ± 5 | 0.9990 | 5.2 | 3.0 | 10 | 1.6 / 4.8 | 3.9 / 3.3 |
| EPB | 2.10 ± 0.03 | 0.01 – 1.00 | 343 ± 5 | 0.9987 | 5.1 | 4.0 | 13 | 1.7 / 7.8 | 4.9 /3.5 |
| PPB | 2.84 ± 0.04 | 0.01 – 1.00 | 306 ± 5 | 0.9984 | 5.0 | 2.0 | 6.7 | 1.7 / 2.3 | 4.3 / 4.2 |
| BzPB | 3.98 ± 0.05 | 0.01 – 1.00 | 270 ± 4 | 0.9989 | 3.7 | 1.0 | 3.3 | 1.6 / 3.3 | 4.2 / 4.1 |
| BP | 5.27 ± 0.04 | 0.10 – 1.00 | 389 ± 6 | 0.9985 | 5.0 | 1.0 | 3.3 | 0.22 / 3.2 | 3.9 / 3.4 |
| BP3 | 6.26 ± 0.04 | 0.10 – 1.00 | 258 ± 5 | 0.9973 | 4.4 | 5.0 | 17 | 5.9 / 7.1 | 1.7 / 3.6 |
| TCS | 7.65 ± 0.06 | 0.10 – 1.00 | 63 ± 2 | 0.9930 | 1.7 | 5.0 | 17 | 3.5 / 8.2 | 5.6 / 6.7 |
| MBC | 9.30 ± 0.05 | 0.10 – 1.00 | 347 ± 7 | 0.9942 | 5.7 | 2.0 | 6.7 | 2.8 / 5.9 | 3.6 / 4.9 |
| OCR | 11.07 ± 0.05 | 0.10 – 1.00 | 119 ± 3 | 0.9937 | 2.6 | 3.0 | 10 | 7.0 / 10 | 2.7 / 6.1 |

[a] Standard deviation associated to the slope
[b] Determination coefficient (8 levels of calibration)
[c] Error of the estimate (standard deviation of the residuals)
[d] Limit of detection calculated (and experimentally verified) as the concentration that provided a signal 3 times the noise
[e] Limit of quantification calculated (and experimentally verified) as the concentration that provided a signal 10 times the noise
[f] Relative standard deviation for 3 experiments in the same day (intra-day) and for 9 experiments in three non-consecutive days (inter-day)

**Table S2**. Experiments of the Doehlert design obtained for the coded matrix of two factors with a hexagonal spatial distribution.

| **Experiment** | **Factor 1** ($t_{desorption}$) | | **Factor 2** ($mL_{methanol}$) | |
|---|---|---|---|---|
| | **C1** | **X1 (min)** | **C2** | **X2 (in mL)** |
| 1 | 0 | 4 | 0 | 1.1 |
| 2 | 0.5 | 5.5 | -0.866 | 0.2 |
| 3 | -0.5 | 2.5 | 0.866 | 2.0 |
| 4 | -0.5 | 2.5 | -0.866 | 0.2 |
| 5 | 0.5 | 5.5 | 0.866 | 2.0 |
| 6 | -1 | 1 | 0 | 1.1 |
| 7 | 1 | 7 | 0 | 1.1 |

$C_1$ and $C_2$ are the coded values for the 5 and 3 levels, respectively
$X_1$ and $X_2$ are the real values of each factor in the experiment

**Table S3**. Results obtained from the analysis of the Doehlert design, where Factor 1 is the desorption time (in minutes) and Factor 2 is the volume of the desorption solvent (methanol, in mL).

| **PCP** | **Optimum value of Factor 1** | **Optimum value of Factor 2** | **Constant** | **Coefficients** | | | | |
|---|---|---|---|---|---|---|---|---|
| | | | | **A** | **B** | **AA** | **AB** | **BB** |
| MPB | 3.7 | 1.257 | 15298 | 32786 | 238 | -3989 | -2.81 | -0.09 |
| EPB | 3.6 | 1.294 | 37150 | 18089 | 196 | -1546 | -5.35 | -0.07 |
| PPB | 7.0 | 0.770 | 105717 | 1631 | 203 | 2641 | -18.33 | -0.05 |
| BzPB | 7.0 | 0.882 | 18644 | 39917 | 316 | -1166 | -24.50 | -0.08 |
| BP | 7.0 | 2.000 | 53538 | 1282 | -2 | -836 | 6.18 | -0.01 |
| BP3 | 4.0 | 1.322 | -8710 | 27698 | 208 | -3013 | -2.67 | -0.07 |
| TCS | 4.2 | 1.174 | -125176 | 114136 | 341 | -13583 | -0.25 | -0.14 |
| MBC | 4.4 | 1.438 | -24881 | 13616 | 299 | -9099 | 46.76 | -0.18 |
| OCR | 1.0 | 2.000 | 793672 | -330717 | -509 | 51200 | -57.72 | 0.39 |

**Table S4**. Precision and recovery studies for the reuse of the MOF CIM-81 as sorbent in the D-µSPE-UHPLC-UV method proposed.

| **PCP** | **Conc. level: 15 µg·L$^{-1}$** | | | **Conc. level: 45 µg·L$^{-1}$** | | |
|---|---|---|---|---|---|---|
| | ***RSD[a] (%)*** | ***RR[b] (%)*** | ***$E_F$[c]*** | ***RSD[a] (%)*** | ***RR[b] (%)*** | ***$E_F$[c]*** |
| MPB | 8.4 | 110 | 8.5 | 8.2 | 102 | 6.3 |
| EPB | 12 | 115 | 6.5 | 13 | 74.9 | 4.6 |
| PPB | 14 | 115 | 8.7 | 11 | 75.5 | 5.9 |
| BzPB | 14 | 113 | 6.8 | 6.4 | 86.7 | 5.5 |
| BP | 15 | 106 | 3.5 | 4.3 | 92.6 | 2.9 |
| BP3 | 4.8 | 91.5 | 5.1 | 4.9 | 75.6 | 4.1 |
| TCS | 6.1 | 129 | 14 | 9.3 | 86.0 | 6.9 |
| MBC | 11 | 94.7 | 2.7 | 7.6 | 81.9 | 2.6 |
| OCR | 14 | 82.9 | 26 | 9.2 | 91.4 | 27 |

[a] Intra-day relative standard deviation (n = 3)
[b] Relative recovery
[c] Enrichment factor
